# Nitrogen metabolic rate and differential ammonia volatilization regulate resistance against opportunistic fungus *Alternaria alternata* in tobacco

**DOI:** 10.3389/fpls.2022.1003534

**Published:** 2022-09-23

**Authors:** Zhixiao Yang, Yi Chen, Yi Wang, Haiqian Xia, Shaoqing Zheng, Shengdong Xie, Yi Cao, Jiemin Liu, Shafaque Sehar, Yingchao Lin, Yushuang Guo, Imran Haider Shamsi

**Affiliations:** ^1^Guizhou Academy of Tobacco Science, Guiyang, Guizhou, China; ^2^Guizhou Provincial People’s Hospital, Guiyang, Guizhou, China; ^3^Zhejiang Key Laboratory of Crop Germplasm Resource, Department of Agronomy, College of Agriculture and Biotechnology, Zhejiang University, Hangzhou, China

**Keywords:** nitrogen, ammonia volatilization, apoplast, correlation analysis, glutamine synthetase, tobacco

## Abstract

Nutritional correlations between plants and pathogens can crucially affect disease severity. As an essential macronutrient, the availability of nitrogen (N) and the types of N content play a fundamental part not only in energy metabolism and protein synthesis but also in pathogenesis. However, a direct connection has not yet been established between differences in the level of resistance and N metabolism. Pertinently, former studies hold ammonia (NH_3_) accountable for the development of diseases in tobacco (*Nicotiana tabacum* L.) and in some post-harvest fruits. With a purpose of pinpointing the function of NH_3_ volatilization on *Alternaria alternata* (Fries) Keissl pathogenesis and its correlation with both N metabolism and resistance differences to *Alternaria alternata* infection in tobacco, leaf tissue of two tobacco cultivars with susceptibility (Changbohuang; CBH), or resistance (Jingyehuang; JYH) were analyzed apropos of ammonia compensation point, apoplastic NH_4_^+^ concentration, pH value as well as activities of key enzymes and N status. At the leaf age of 40 to 60 d, the susceptible cultivar had a significantly higher foliar apoplastic ammonium (NH_4_^+^) concentration, pH value and NH_3_ volatilization potential compared to the resistant one accompanied by a significant reduction in glutamine synthetase (GS), which in particular was a primary factor causing the NH_3_ volatilization. The NH_4_^+^ concentration in CBH was 1.44 times higher than that in JYH, and CBH had NH_3_ compensation points that were 7.09, 6.15 and 4.35-fold higher than those of JYH at 40, 50 and 60 d, respectively. Moreover, the glutamate dehydrogenase (GDH) activity had an upward tendency related to an increased NH_4_^+^ accumulation in both leaf tissues and apoplast but not with the NH_3_ compensation point. Collectively, our results strongly suggest that the accumulation of NH_3_ volatilization, rather than NH_4_^+^ and total N, was the primary factor inducing the *Alternaria alternata* infection in tobacco. Meanwhile, the susceptible cultivar was characterized by a higher N re-transfer ability of NH_3_ volatilization, in contrast to the disease–resistant cultivar, and had a stronger capability of N assimilation and reutilization. This study provides a deeper understanding of the pathogenicity mechanism induced by *Alternaria alternata,* which is useful for breeding *Alternaria alternata*-resistant varieties of tobacco, at the same time, our research is also conducive to control tobacco brown spot caused by *Alternaria alternata* in the field.

## Introduction

Nutrient elements such as nitrogen (N) can profoundly affect disease development, and the expression of certain pathogenicity–related genes and virulence/avirulence responses are also altered by the host plant’s N status (Snoeijers et al., 2000; Sun et al., 2021). Successful plant colonization by pathogen requires the utilization of nutrient resources present in host tissues, and overcoming this challenge becomes easier when plants contain adequate nutrition (Mu et al., 2000; Ballini et al., 2013). However, various nutritional limitations, in particular N, also tend to influence pathogenesis (Yu et al., 2018). The observation that both fungal and bacterial genes are induced in regard to N deficiency in artificial media infers that the utilization of N by pathogen should be limited during growth *in planta* (Talbot et al., 1997). Nevertheless, the presence of foliar soluble N in millimolar concentration (Farrar, 1995), even under the condition of N deficiency in the plant (López-Berges et al., 2010), apparently contradicts the idea of pathogen having access only to a specific subcomponent of soluble N pool (Walters and Bingham, 2007). For most interactions involving plants and pathogens, little information is available regarding the composition and content of N during infection and subsequent colonization. Furthermore, a direct link has not yet been established between N–shortage stress and pathogen virulence (Snoeijers et al., 2000).

According to Walters and Bingham (2007), pathogen invasion involves encountering a series of different forms of N in the symplast and apoplast of plant tissues, ranging from inorganic N (e.g., nitrate), to organic N (e.g., glutamine). Ammonia is readily adsorbed onto wet leaf cuticle surface, contributing to the main pathway of N loss from plant leaves (Sparks, 2009). Lots of previous studies have indicated that NH_3_ is involved in plant-pathogen interactions. An important influencing factor for the expansion of *Erwinia carotovora* in potato is NH_3_ accumulation (Lowekovich et al., 1967). Huber and Watson (1974) found that NH_3_ could stimulate diseases caused by *Fusarium oxysporum*, *Rhizoctonia solani* and *Sclerotinia sclerotiorum* on citrus, cotton, sugar beet, tomato and wheat. The host’s NH_3_ signal also shares a key role in the infection caused by post-harvest pathogenic fungi such as *Alternaria alternata* and *Colletotrichum gloeosporioides* (Prusky et al., 2001; Prusky and Yakoby, 2003; Alkan et al., 2008; Prusky and Lichter, 2008). The buildup of NH_3_ at the site of infection during the decomposition of avocado fruits portrays a specific condition that is perceived by the pathogen; NH_3_ directly triggers the expression of pathogenicity factors in *Colletotrichum gloeosporioides*, such as *PELB* which encodes for pectate lyase (Kramer-Haimovich et al., 2006). Pathogens can even alter pH around the infection site, which in turn modulates the action of pathogenicity factors (Prusky et al., 2001; Eshel et al., 2002; Prusky and Yakoby, 2003). In a study on *Alternaria alternata* (Fries) Keissl causing brown spot disease, which accelerates senescence in tobacco leaves, the facultative parasitic fungus responded to ambient NH_3_ and used it as a stimulator to attack host by differentiating into infection structures, switching to a necrotrophic lifestyle (Duan et al., 2010). Furthermore, at the status of 10^−6^–10^−4^ mol L^−1^ NH_3_, the infection of *Pseudomonas syringae* pv. *tabaci* was elevated, suggesting that an appropriate NH_3_ level could promote pathogenicity (Li, 2018). However, it is worthwhile to note that, fungal pathogens are predisposed to a range of fluctuations in the NH_3_ environment of their host, attributable to the leaf senescence that correlates with both NH_3_ and NH_4_^+^ accumulation in the foliar tissues and apoplast (Sutton et al., 1993, 1998; Masclaux et al., 2000; Schjoerring et al., 2002). Furthermore, nitrate (NO_3_^−^) has been found to increase disease resistance to *Pseudomonas syringae* pv. phaseolicola and *Fusarium oxysporum* in tobacco and cucumber, respectively (Gupta et al., 2013; Sun et al., 2021). However, few data are available regarding *in planta* differences in NH_4_^+^ concentrations between susceptible and resistant cultivars.

Different tobacco cultivars have diverse N metabolism reactions (Duan et al., 2012; Yang et al., 2015; Wu et al., 2016). Relatedly, high incidence rate of *Alternaria alternata*–mediated brown spot disease is always associated with sufficient N in tobacco plants (Stavely and Main, 1970). In the process of growth and development, plants can lose N in the form of NH_3_, with the consequence that NH_3_ exchange occurs between plants and external environment, and this volatilization of NH_3_ is strongest in the leaf senescence stage (Massad et al., 2008; Chen et al., 2009; Herrmann et al., 2009). The apoplast is considered to be the primary reservoir of NH_4_^+^ and its concentration in the apoplast has an important effect on the volatilization of NH_3_ in plants (Nielsen and Schjoerring, 1998). The balance between NH_3_ and NH_4_^+^ in the apoplast could be achieved by NH_3_ volatilization (Jiang, 2017); moreover, the NH_4_^+^ in the apoplast is very sensitive to leaf N status and its external supply (Schjoerring et al., 2000). Noteworthily, the apoplast is a key part where early interaction occurs between host plant and pathogen upon pathogen infection, and the exchanges of NH_3_ between plants and atmosphere also happen through the apoplast (Hammond-Kosack and Parker, 2003; Herrmann et al., 2009). In particular, as a unity of structure and function, the apoplast plays important roles pertinent to the instigation and co-ordination of certain defense responses (Yakimova et al., 2009); for instance, the reactive oxygen species (ROS) can move into the apoplast, and directly act on the invading pathogen (Wilkinson and Davies, 1997; Bolwell et al., 2001). Based on these reasons, deeply clarifying the role of apoplastic NH_3_ volatilization in pathogenicity is valuable for revealing the inducing factors of *Alternaria alternata* from quiescent biotrophic growth to necrotrophic stage.

Tobacco is an important economic plant in China and the principal production areas are concentrated in remote rural areas with less developed economies, such as Yunnan, Guizhou, etc. However, it is easily susceptible to *Alternaria alternata* infection, which adversely affects the yield and quality, but until now there is no effective prevention method (Slavov et al., 2004). In fact, the effect of N nutrition depends on plant–pathogen interactions, in particular, the pathogenic lifestyle and pathosystems (Dordas, 2008). To our knowledge, fewer studies have examined the differences in the N status and metabolic reactions in tobacco cultivars that are resistant and/or susceptible to *Alternaria alternata* infection. Products of N metabolism also function as signaling molecules to trigger defense responses, following pathogen recognition and signal transduction processes (Kachroo and Robin, 2013; Rojas et al., 2014; Thalineau et al., 2016). Given the importance of N metabolism in agriculture, in this study, we investigated the function of NH_3_ volatilization as regulated by the apoplast in pathogenesis and its correlation with N metabolism and resistance differences to pathogens infection. Keeping the aim in view, parameters such as ammonia compensation point, apoplastic NH_4_^+^ concentration, pH value, the contents of total N, soluble protein and NH_4_^+^ concentration in leaf tissue, as well as N metabolism related key enzymes activities were measured in two cultivars with resistance or susceptibility to *Alternaria alternata.* The study provides further insights into the role of NH_3_ volatilization in the pathogenicity mechanism induced by *Alternaria alternata* and delivers a deeper understanding of the metabolic basis of resistance.

## Materials and methods

### Experimental materials and growth conditions

Differing in their response to *Alternaria alternata* attack, two tobacco cultivars, i.e., Jingyehuang (JYH, resistant) and Changbohuang (CBH, susceptible) provided by Guizhou academy of tobacco science, were used as the experimental materials. Moreover, JYH was bred by system selection from disease-resistant individuals of CBH, which is used as core parent for breeding brown spot-resistant varieties in tobacco (Yang et al., 2018). The loamy textured soil used in this study contained 8.50 g kg^−1^ organic matter, 0.89 g kg^−1^ total N, 0.07 g kg^−1^ available N, 0.03 g kg^−1^ available phosphorus (P), and 0.11 g kg^−1^ available potassium (K), with a pH of 7.91. Additionally, the soil was air-dried and sterilized by fumigation, before passing through a 0.5 × 1 cm screen.

The tobacco seeds were sterilized with a 0.2% CuSO_4_ (w/v) solution for 10 min, and then washed with deionized water. The seeds were allowed to germinate in floating trays consisting of 20% vermiculite, 70% peat and 10% perlite (v/v), and the nutrition for tobacco seeds growth was supplied with modified Hogland nutrient solution (10 mol L^−1^ KNO_3_, 1 mol L^−1^ CaCl_2_, 1 mol L^−1^ MgSO_4_, 2 mol L^−1^ KH_2_PO_4_, 200 μmol L^−1^ EDTA-Fe, and 0.5 ml L^−1^ microelements buffered with 0.5 g L^−1^ MES [2-(*N*-morpholino) ethanesulfonic acid), pH 5.5; Hu et al., 2019] and grown in a greenhouse with day-night temperatures of 28 ± 2°C and 25 ± 2°C, respectively. The relative humidity of air (RH) was maintained around 70 ± 5% under a 16–h photoperiod (light intensity >400 μmol m^−2^ s^−1^). The seedlings (55 d post-germination), averaging approximately 12 cm in height, were transplanted into polyethylene plastic pots (27 × 33 × 21 cm, height × caliber × bottom diameter) with a load of 20 kg of soil and grown under conditions mentioned above. Each pot contained only one plant, 80 plants per cultivar were used. The experiment was conducted with a completely random design with three replicates. At the end of experiment, the tested soil contained 0.2 N, 0.4 P and 0.5 K (g nutrient kg^−1^ soil) after fertilization with NH_4_^+^–N (1.36): NO_3_^−^–N (1.14), taking NH_4_NO_3_, NaH_2_PO_4_ and K_2_SO_4_ as fertilizer source. The samples were collected (13th leaf from the bottom of tobacco plant) in the late morning (9.30–11.30 a.m.) at 30, 40, 50, 60, and 70 d (days after leaf sprouting) of leaf age, which paralleled the leaf expansion stage (before 40 d), ripening and senescence period (after 40 d), respectively. Counting of leaf age began from the first day when the sprout was 1 cm long and 0.5 cm wide, and along with it the leaf labeling began. The approximate time of flowering was around 40 d in this study. All sampled leaves were washed with distilled water, immediately frozen in liquid nitrogen and stored at −80°C until further analysis.

### Measurements

#### Apoplastic fluid and leaf tissue extraction

For the extraction of apoplastic fluid, a vacuum infiltration technique was employed (Husted and Schjoerring, 1995). In case of leaf tissue extraction, samples were homogenized in 10 mmol L^−1^ of formic acid in a cooled mortar with a little sand, then centrifuged at 25,000 × *g* (2°C) for 10 min, followed by transference of supernatant to a 0.45–μm polysulphone centrifugation filter (Micro Vectra Spin, Whatman Ltd., Maidstone, United Kingdom), and spinning at 5,000 × *g* (2°C) for 5 min.

#### Analyses of GS, GDH activities, apoplastic NH_4_^+^ concentration and pH value

Activity of glutamine synthetase (GS) was tested according to the method of Meng et al. (2016) by measuring formed γ-glutamyl-hydroxamate, while a protocol described by Turano et al. (1996) was followed for glutamate dehydrogenase (GDH) assay based on the measurement of decrease or increase in absorbance of samples (respective of the direction of the reaction) at 340 nm using a spectrophotometer *Novaspec II* (*Pharmacia*, Uppsala, Sweden). The apoplastic NH_4_^+^ concentration was determined with a continuous flowing analyzer (Bran Luebbe AA3), using calibration solutions depending on the concentration of the samples, i.e., 0.1 and 1.0 μg NH_4_^+^ kg^−1^ or 1.0 and 10 μg NH_4_^+^ kg^−1^ (NH_4_Cl in ddH_2_O). Deionized water was used as zero standard. Moreover, the pH value was monitored directly in the micro-centrifuge tube by inserting a microelectrode (Mettler Toledo, Inlab 423, Electroly, 9,811).

#### Quantification of different N compounds in leaf tissues

The NH_4_^+^ concentration in leaf tissue was examined through fluorimetry using an HPLC system (Waters Corp., Milford, MA, United States) fitted with a pump, a column oven with a 3.3-m stainless steel reaction coil, an autosampler cooled to 2°C and a scanning fluorescence detector. The reaction took place between NH_4_^+^ and *o*-phthalaldehyde to form an alkylthioisoindole fluorochrome at neutral pH with β-mercaptoethanol (as reducing agent). Excitation and emission wavelengths were 410 and 470 nm, respectively (O’Leary et al., 2014). Carlo Erba Model-1,106 Elemental Analyzer (Carlo Erba, Milan, Italy) was employed to determine total N content in leaf tissues (Horneck and Miller, 1998). Soluble protein content in the crude leaf extracts (the same as used for GS activity) were detected by a protein assay kit (Bio-Rad, Munich, Germany), keeping bovine serum albumin as standard.

### Statistical analysis

All the measurements were conducted with three independent biological replicates for each determination, and mean values are presented with standard errors. Data were analyzed with SPSS-17.0 (statistical software package) using one-way ANOVA, while least significance difference (LSD) test at 0.05 probability assisted in separating differences in means according to the method of Hosseini et al. (2022).

## Results

### Concentration of apoplastic NH_4_^+^ and pH value

**Figure 1:**
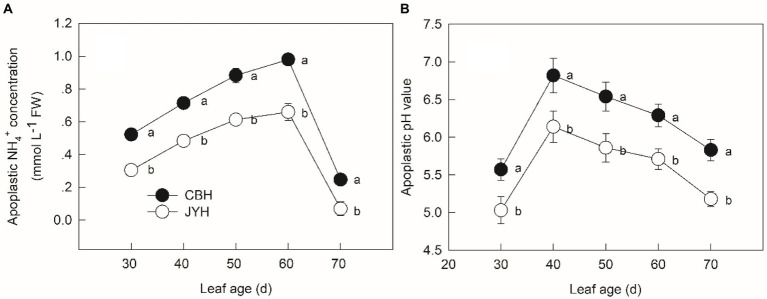
Apoplastic NH_4_^+^ concentration **(A)** and pH value **(B)** in resistant and susceptible tobacco cultivars to *Alternaria alternata*. Values with different letters are significantly different (*p* ≤ 0.05).

The NH_4_^+^ concentration in apoplastic solution increased with leaf age except for 60 to 70 d, the maximum concentration in CBH was 1.44 times larger than that in JYH (Figure 1A). Significantly higher apoplastic NH_4_^+^ concentration was observed for CBH compared to JYH, keeping all the leaf collection stages in perspective (*p* ≤ 0.05). For both tobacco cultivars, there was a rise in apoplastic pH value in the initially obtained samples (leaf age from 30 to 40 d), which consecutively decreased at the stage of 40 to 70 d (Figure 1B). The pH values elevated by more than 1 unit in CBH, which occurred much rapidly than that in JYH, and exceeded 6.0. Consequently, those values remained higher in CBH than in JYH during the entire period (*p* ≤ 0.05).

### Potential for NH_3_ volatilization

**Figure 2:**
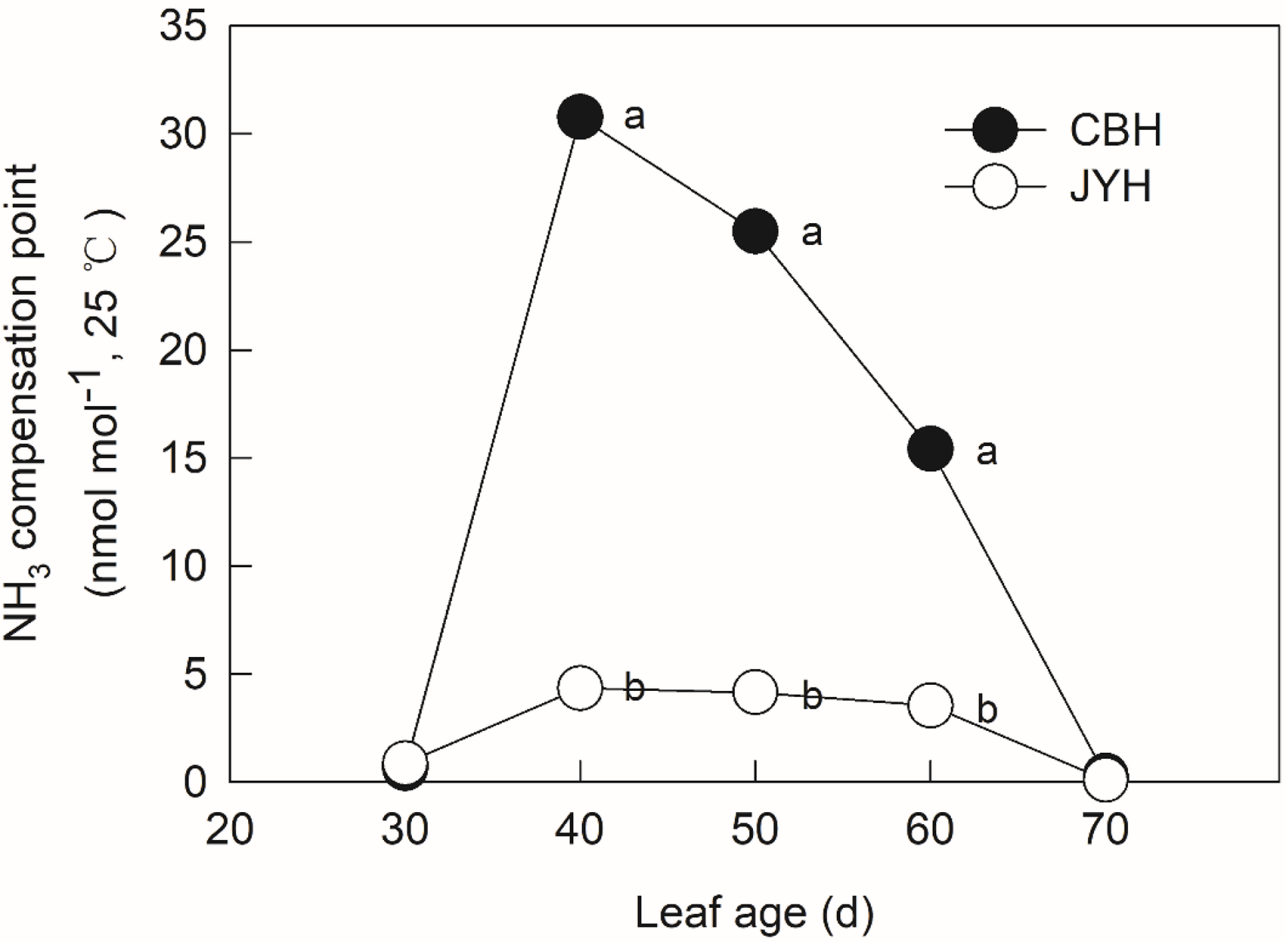
NH_3_ compensation point in resistant and susceptible tobacco cultivars to *Alternaria alternata*. Values with different letters are significantly different (*p* ≤ 0.05).

The potential for NH_3_ loss from the senescing plant material was evaluated by calculating the ratio between [NH_4_^+^] and [H^+^], which is a temperature-independent value, termed as NH_3_ compensation point, utilizing the apoplastic solution. The trend ascended significantly in the initially obtained samples of both cultivars (Figure 2). The increase indicated that tobacco plant lost N through NH_3_ volatilization due to NH_3_/NH_4_^+^ accumulation in leaf tissues and apoplastic solution. Moreover, CBH had NH_3_ compensation points that were 7.09, 6.15 and 4.35-fold higher than those of JYH at 40, 50 and 60 d, respectively. Although the apoplastic pH value decreased from 50 to 60 d, the NH_3_ compensation point still remained higher due to the increase in apoplastic NH_4_^+^ concentration.

### Concentration of NH_4_^+^, total N and soluble protein in leaf tissues

**Figure 3:**
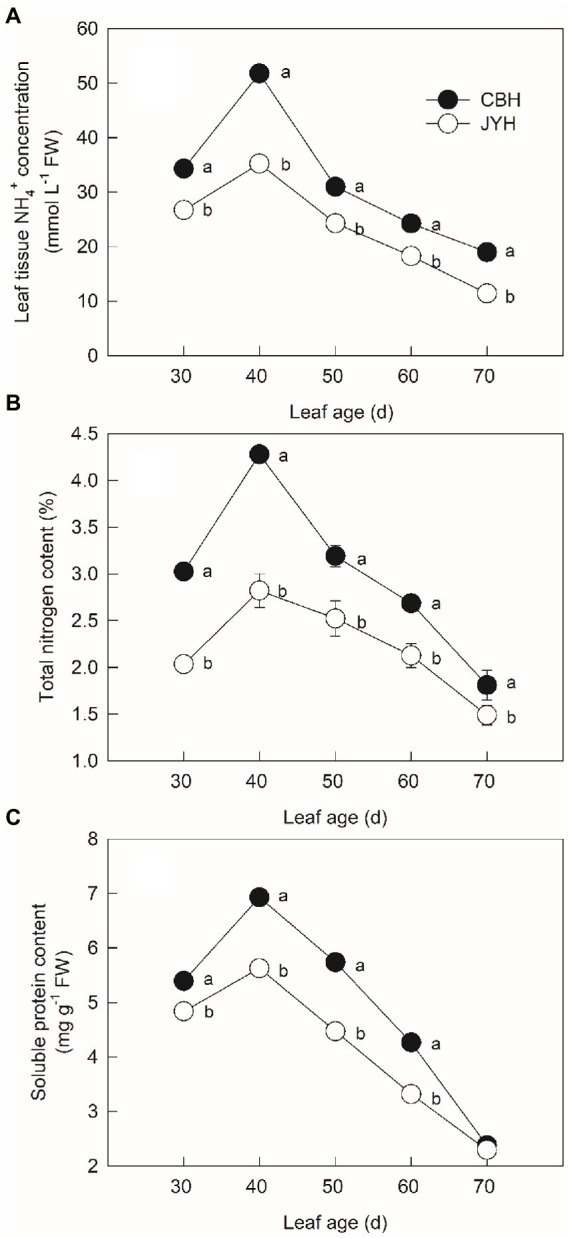
Leaf tissue NH_4_^+^ concentration **(A)** and contents of total nitrogen **(B)** and soluble protein **(C)** in resistant and susceptible tobacco cultivars to *Alternaria alternata*. Values with different letters are significantly different (*p* ≤ 0.05).

Based on the results indicated in Figure 3A, the leaf NH_4_^+^ concentration portrayed an upward trend in two cultivars from 30 to 40 d leaf age. During the period of 40 to 70 d, the furtherance of senescence resulted in a pronounced and continuous decrease in the leaf tissue NH_4_^+^ concentration. Through the course of experiment, the leaf tissue NH_4_^+^ concentration in these cultivars was CBH > JYH; also, CBH showed much rapid increase in the leaf tissue NH_4_^+^ concentration than that of JYH. The total N content in leaf tissue was in the form of successively rising with the developmental stage between 30 and 40 d in these two cultivars and thereafter declined progressively (Figure 3B). Higher total N content was found in CBH than that in JYH throughout the experimental period (*p* ≤ 0.05). At the last stage of 70 d, the total N content decreased 43.47 and 56.74% from the highest values in CBH and JYH, respectively. Altogether, the content of soluble protein in JYH and CBH had similar variation throughout the temporal phases of experiment, with both showing a gradual decline, and the reductions were 53.81 and 59.64%, respectively (Figure 3C). At the stage from 30 to 60 d, CBH had higher soluble protein content than JYH (*p* ≤ 0.05), on the contrary, the two genotypes did not differ significantly at leaf stage of 70 d.

### Glutamine synthetase and glutamate dehydrogenase activity assays in leaf tissue

**Figure 4:**
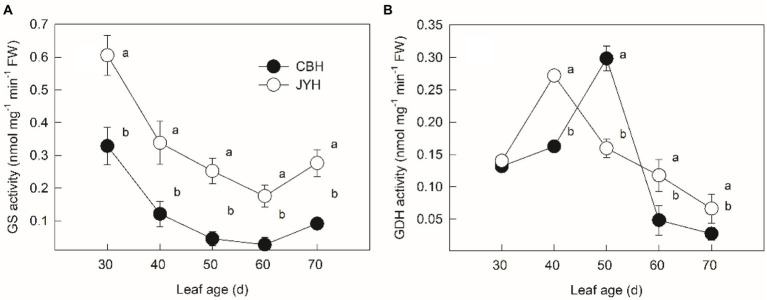
Glutamine synthetase (GS) **(A)** and glutamate dehydrogenase (GDH) **(B)** activities in leaf tissue in resistant and susceptible tobacco cultivars to *Alternaria alternata*. Values with different letters are significantly different (*p* ≤ 0.05).

As depicted in Figure 4A, GS activities dwindled markedly in both cultivars during the period from 30 to 60 d, whereas an upward tendency was observed when reaching the stage of 70 d. A continuous display of significantly higher GS activity was observed for JYH in comparison to CBH throughout the experimental stages (*p* ≤ 0.05). According to Figure 4B, GDH activities in both JYH and CBH had a pronounced and gradual decrease following an increase in the beginning stages of leaf age samples. However, more than 10 days of increase was observed in CBH, and as a consequence, GDH activity was significantly higher in CBH than that in JYH at 50 d (*p* ≤ 0.05).

### Correlation analysis of N metabolism-related parameters

**Figure 5:**
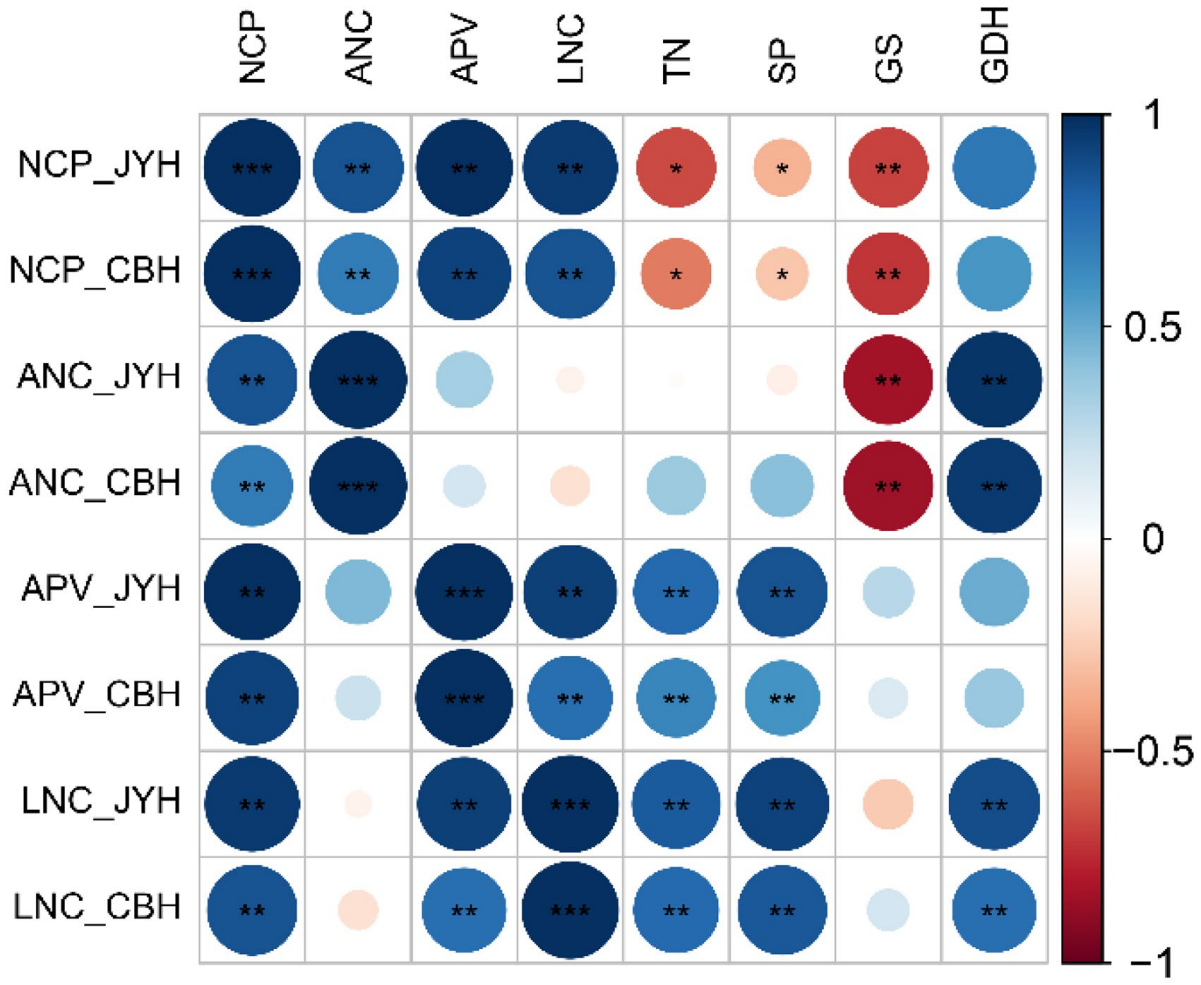
Correlation analysis of nitrogen metabolism–related parameters in resistant and susceptible tobacco cultivars to *Alternaria alternata.* Abbreviations: NH_3_ compensation point–NCP. Apoplastic NH_4_^+^ concentration–ANC. Apoplastic pH value–APV. Leaf tissue NH_4_^+^ concentration–LNC. Total nitrogen content–TN. Soluble protein content–SP. GS activity–GS. GDH activity–GDH. *Significant at *p* ≤ 0.05 level. ^**^Significant at *p* ≤ 0.01 level.

Correlation analyses of N metabolism-related parameters in JYH and CBH were performed, and a significance test was also conducted. On the basis of present results showed in Figure 5 and Table 1, a highly significant negative correlation was observed between apoplastic NH_4_^+^ concentration and GS activity in these two tobacco cultivars. By contrast, apoplastic pH value had a significant or highly significant positive correlation with the total N contents and soluble proteins in foliar tissues, also a very positive correlation with leaf tissue NH_4_^+^ concentration was exhibited. Simultaneously, there existed a highly significant positive correlation between the NH_4_^+^ concentrations and GDH activity in the leaf tissues and apoplast. However, the NH_3_ compensation point was independent of GDH activity. The results revealed that the NH_3_ compensation point and GS activity had a highly significant inverse correlational dependence, in addition to a remarkable negative correlation with the contents of total N and soluble protein in leaf tissue. Nevertheless, the NH_3_ compensation point had substantial positive correlation with apoplastic pH value and NH_4_^+^ concentration in both leaf tissues and apoplast. Meanwhile, the correlation analysis also displayed that the NH_3_ compensation point had an upward trend in a situation where GS activity and the contents of total N and soluble protein in leaf tissues decreased while apoplastic NH_4_^+^ concentration and pH value both increased, and which ultimately led to an elevation in NH_3_ volatilization.

**Table 1:** Correlation analysis of nitrogen metabolism–related parameters in resistant and susceptible tobacco cultivars.

**Parameters**	**Cultivar**	**NH**_**3**_ **compensation point**	**Apoplastic NH**_**4**_^**+**^ **concentration**	**Apoplastic pH value**	**Leaf tissue NH**_**4**_^**+**^ **concentration**	**Total nitrogen content**	**Soluble protein content**	**GS activity**	**GDH activity**
**NH**_**3**_ **compensation point**	JYH	1.00	0.86^**^	0.99^**^	0.95^**^	−0.66^*^	−0.34^*^	−0.67^**^	0.71
	CBH	1.00	0.69^**^	0.92^**^	0.86^**^	−0.52^*^	−0.28^*^	−0.72^**^	0.58
**Apoplastic NH**_**4**_^**+**^ **concentration**	JYH	0.86^**^	1.00	0.33	−0.07	−0.02	−0.09	−0.84^**^	0.97^**^
	CBH	0.69^**^	1.00	0.18	−0.16	0.36	0.41	−0.85^**^	0.95^**^
**Apoplastic pH value**	JYH	0.99^**^	0.44	1.00	0.93^**^	0.77^*^ ^*^	0.86^**^	0.27	0.49
	CBH	0.92^**^	0.21	1.00	0.75^**^	0.65^**^	0.59^**^	0.16	0.37
**Leaf tissue NH**_**4**_^**+**^**concentration**	JYH	0.95^**^	−0.07	0.93^**^	1.00	0.83^**^	0.92^**^	−0.26	0.88^**^
	CBH	0.86^**^	−0.16	0.75^**^	1.00	0.77^**^	0.84^**^	0.18	0.75^**^

* *Significant at the p ≤ 0.05 level.*

** *Significant at the p ≤ 0.01 level.*

## Discussion

Nitrogen plays an essential role in plant–pathogen interactions (Wang et al., 2019). Depending on the plant species and pathogen strains, N can affect the resistance and susceptibility of a plant to diseases by regulating plant growth and physiology, influencing pathogen growth and virulence, and modifying the rhizosphere environment (Fagard et al., 2014). Previous reports have inferred that the invasion on tobacco from *Alternaria alternata* escalates with increasing leaf N content (Stavely and Main, 1970). Moreover, N availability is deduced as a regulatory factor for the phytopathogenic fungal colonization (Stephenson et al., 1997; Talbot et al., 1997; Snoeijers et al., 2000). Excessive N supply has been registered as useful particularly for the sporulation capacity of colonies and the cumulative production of spores on leaves (Jensen and Munk, 1997; Robert et al., 2005). A further link between host and pathogen N was found by Robert et al. (2002) who correlated spore production by the fungus *Puccinia triticina* in wheat. The spores number was 70% less in the low N plants but the percentage of N in the spores was higher than in the leaves, suggesting that the pathogen is highly efficient at taking up N from the host would be important for virulence. Consistent with previous scientific literature, the close relationship between N utilization capacity and susceptibility of tobacco cultivars to *Alternaria alternata* infection in the present work demonstrated that higher content of total N was detected in CBH (susceptible cultivar) than that in the disease–resistant one, i.e., JYH. Therefore, the disease–resistant cultivar apparently had lower N availability. Furthermore, the NH_4_^+^ concentration in these two cultivars concurred with their resistance to *Alternaria alternata* infection. Moreover, the changes in leaf tissue NH_4_^+^ concentration and apoplastic pH value were also concomitant, and so were the consequent calculated NH_3_ compensation point.

During host-pathogen interactions, pathogen and host compete for N–based nutrients, and the pathogen may affect the mobilization and distribution of N in the host plants so as to meet its own demands for growth (Pageau et al., 2006; Jedelská et al., 2021). Increased supply of N in the plants led to higher spore production by the powdery mildew fungus *Oidium lycopersicum*, and the increment in leaf colonization by the bacterium *Pseudomonas syringae* pv tomato suggested that increased leaf N caused greater susceptibility to these pathogens (Mur et al., 2016). In the present research, it was found that the susceptible cultivars had a higher total N content than the disease–resistant cultivar. Brown spot disease is known to be favored by excess N (Stavely and Main, 1970). After tobacco leaves enter a mature stage, *Alternaria alternata* begin to infect and colonize the tissues. The higher total N content in the susceptible cultivar provides more nutrients to this fungal pathogen, which is beneficial to the extension of mycelium and sporulation. The lower N level in the disease–resistant cultivar may have resulted in nutrient demands of *Alternaria alternata* not being met limiting infection and spread. This variation in N content between disease–resistant and susceptible cultivars may be an important factor responsible for the differences between the cultivars in their resistance to *Alternaria alternata* infection (Barrit et al., 2022).

As the results of this research revealed, the susceptible cultivar had significantly higher concentration of apoplastic NH_4_^+^ and pH value, as well as the NH_3_ compensation point than those in the disease–resistant cultivar from 40 to 60 d, in the course of which the volatilization of NH_3_ appeared to be significant. Duan et al. (2010) found that *Alternaria alternata* could sense ambient NH_3_, get stimulated by it for invasion and prompts infection structures’ differentiation on tobacco leaves, finally accelerating a shift from biotrophic process to a lifestyle contingent on necrotrophy, by the secretion of pathogenicity factors. Additionally, *Alternaria alternata* and *Glomerella cingulata* in fruits make use of the changes of N status in host and even actively secrete NH_3_ to alkalize the host tissue, which ensues the host parasitic to saprophyte transformation (Eshel et al., 2002; Prusky et al., 2006). Consecutively, the gradients of leaf pH value also affect the growth direction of germ tube in *Uromyces viciae*-*fabae* (Edwards and Bowling, 1986). In addition, our current results exhibited that the susceptible cultivar had greater increase in the apoplastic pH value and NH_3_ compensation point as compared to the disease–resistant one, with a higher accumulation of NH_4_^+^ concentrations in the leaf tissue and apoplast, which resulted in greater NH_3_ volatilization (Walker et al., 2012). In particular, NH_4_^+^ has been reported to increase resistance against *Pseudomonas*. *syringae* in tomato (González-Hernández et al., 2019). Eventually, high level of NH_3_ shifts in host could produce an appropriate condition, which is helpful for the *Alternaria alternata* infection. On the contrary, the disease–resistant cultivar had smaller N metabolism-related parameters and N status as opposed to the susceptible one, and as a consequence, the NH_3_ volatilization was lower. In this sense, these variations in the NH_3_ environment and apoplastic pH value might not be sufficient to cause the infection reaction of *Alternaria alternata* on the disease–resistant tobacco cultivar. To deal with pathogens invasion, plants have evolved sophisticated defence mechanisms, including inducible and constitutive resistance mechanisms. Contrary to the constitutive defence, which typically consists of physical barriers and pre-formed chemical compounds, the pathogen-induced plant resistance depends on the activation of downstream defence responses (Sun et al., 2020). Our research results reinforce the role of NH_3_ accretion in the invasion of susceptible cultivar by *Alternaria alternata*.

Increment in the apoplastic pH value resulted from NH_3_/NH_4_^+^ accumulation in the leaf tissues and apoplastic fluid, displaying a consumption of protons with the excretion of NH_3_/NH_4_^+^ from leaf cells into apoplast (Wang et al., 2013), and in most plant species the apoplastic pH values usually ranges from 5.0 to 6.5 (Husted and Schjoerring, 1995; Mattsson et al., 1997). However, the transformation from living parasite phase to saprophytic stage in facultative pathogenic fungi during fruit ripening is closely related to the rise in pH and NH_4_^+^ concentration. For polyphagous pathogens such as *Alternaria alternata*, a three- to ten-fold increase in NH_3_ concentration and a 0.2 to 2.4 units of pH elevation are detected in several hosts, including cherry, melon, persimmon, pepper and tomato (Eshel et al., 2002). The expression of *AAK1* (an endoglucanase gene) in *Alternaria alternata* is maximal at pH value beyond 6.0, which is a characteristic of decaying tissue, whereas at lower pH condition neither this gene is expressed nor the pathogen is active. The previous study also reported that for mycelial growth and/or sporulation of *Alternaria alternata*, the optimal pH value was between 6.0 and 8.0 (Du et al., 2009). In our present research, the susceptible cultivar showed pH values greater than 6.0 during senescence, compared with those in the disease–resistant one. Currently, no data are available about the pathogenesis, and therefore, obtaining more knowledge regarding the effects of pH value on infection by fungus, particularly in the senescence stage, is important.

Most plants use inorganic nitrogen, NO_3_^−^ and NH_4_^+^, as their primary N source (Soulie et al., 2020). Several reactions occur in plant tissues can release NH_4_^+^ from organic compounds and among those, the most important NH_4_^+^ production processes are ammonium uptake through roots, nitrate reduction, deamination, photorespiration and protein degradation during senescence (Joy, 1988). According to Leegood et al. (1995), this NH_4_^+^ should be re-assimilated to prevent plant from being depleted of N, because the photorespiratory NH_4_^+^ release may take place at ten-fold higher rates compared to the rates of primary NH_4_^+^ assimilation. Correspondingly, a key enzyme GS re-assimilates NH_4_^+^, which is not only involved in the regulation of NH_4_^+^ concentration in plant tissues (Schjoerring et al., 2002), but also the flux of NH_3_ between plant and atmosphere (Husted and Schjoerring, 1995). The genotypes with lower GS activity are able to exhibit higher NH_3_ volatilization (Husted and Schjoerring, 1996; Mattsson et al., 1997; Husted et al., 2002). In the present experiment, the rise in apoplastic pH, NH_3_ compensation point and leaf tissue NH_4_^+^ concentration occurred simultaneously, accompanied by a decrease in GS activity. However, the response of the susceptible cultivar was steeper than that in the disease–resistant one. Meanwhile, compared with the disease–resistant cultivar, higher NH_4_^+^ concentration and a more rapid increase in pH value in the apoplast were detected in the susceptible one, most likely due to insufficient GS activity for the re-assimilation of higher quantities of NH_3_ released during leaf senescence by protein degradation. By contrast, the higher GS activity in the disease–resistant cultivar ensured that the NH_4_^+^ concentration in leaf tissue did not accumulate to a harmful level, but was stronger in N assimilation and reutilization; nitrogen was thus less volatilized in NH_3_ (Hao et al., 2020). On this basis, the obvious distinctions in N metabolism among various tobacco cultivars are the decisive factors for the differential NH_3_ compensation points.

In our work, the promotion of **NH**_**4**_^**+**^ concentration in the two tobacco cultivars with differing resistance was indeed concomitant with both the increase in GDH activity and the dramatic decline in GS activity. Although, GDH is induced along with a buildup of **NH**_**4**_^**+**^ resulting from hydrolysis of proteins during natural leaf senescence (Masclaux et al., 2000), its function in higher plants remains debatable. For example, the upregulation of GDH activity in response to elevated levels of **NH**_**4**_^**+**^ imply its importance in the detoxification of **NH**_**4**_^**+**^ by assimilating some of the excess **NH**_**4**_^**+**^ ions (Terce-Laforgue et al., 2004a; Tercé-Laforgue et al., 2004b). However, GDH also partakes in the deamination process by converting amino acids (AAs) into transport compounds with a lower C/N ratio, for instance, in senescing leaves and germinating seeds (Frechilla et al., 2002). Additionally, this concept is fully backed by a number of experiments employing tobacco plants (Masclaux-Daubresse et al., 2006; Purnell and Botella, 2006; Skopelitis et al., 2007). Labboun et al. (2009) documented the suppression of glutamate synthesis in the presence of NH_4_^+^ in transgenic tobacco leaves when GDH was inhibited. The current experimental results also indicated that the GDH activity significantly and positively correlated with the NH_4_^+^ concentrations in the apoplast and leaf tissues, which inferred that the increase in GDH activity could contribute to the elevated NH_4_^+^ concentration. Similarly, rice mutants lacking *Fd*-*GOGAT* have enhanced resistance to seven *Xanthomonas oryzae* pv. *oryzae* (*Xoo*) strains (Chen et al., 2016). It seems that N-related key enzymes are affected by pathogen, and the activity modification can alter N metabolism, leading to remarkable effects on disease development. Thus, how the N metabolism are associated with plant defence and the underlying mechanisms need to be intensive tested in a biological context using the same system, which might account for the highly specific nature of tobacco–*Alternaria alternata* interactions in response to N conditions.

## Conclusion

In conclusion, during the leaf age of 30 to 70 d, the N metabolism–related parameters including NH_4_^+^ concentrations in the leaf tissue and apoplast, apoplastic pH value, NH_3_ compensation point and N status in the resistant cultivar were all lower in comparison with the susceptible one, and eventually led to a weaker ability of NH_3_ volatilization. In contrast, the susceptible cultivar had superior NH_3_ volatilization potential against the disease–resistant cultivar, which precisely created a favorable environment for the instigation of *Alternaria alternata* infection. In addition, the apoplast actively regulated the exchange between NH_3_ and external environment, and the NH_3_ volatilization primarily resulted from a sharp reduction in GS activity. Simultaneously, the GDH activity surge was positively related to a rise in the accumulation levels of NH_4_^+^, whereas the activity was independent of the NH_3_ compensation point. The results also revealed that there were lower GS activity and higher NH_3_ compensation point in the susceptible cultivar, which could give rise to more NH_3_ volatilization. On the contrary, the disease–resistant cultivar had higher GS activity and a lower NH_3_ compensation point and primarily depended on the ability of N assimilation and reutilization to remove NH_4_^+^ accumulation. The present research gave new insights into the connection between N metabolism, especially the function of NH_3_ volatilization and the resistance differences to *Alternaria alternata*, indicating that N metabolism is in a close association with the resistance or susceptibility to *Alternaria alternata* attack. However, N metabolism is a complex process, which can be affected by many factors, and the N content, leaf tissue and apoplastic NH_4_^+^ concentrations, apoplastic pH and key enzyme activities do not fully reveal the resistance mechanism against *Alternaria alternata*. Many other factors, such as special physiological and biochemical processes may be at play and still need to be further clarified.

## Data availability statement

The original contributions presented in the study are included in the article/Supplementary material; further inquiries can be directed to the corresponding authors.

## Author contributions

YL, YG, and IS designed the research. ZY, YW, HX, SZ, and SX performed the research. ZY and YC analyzed the data. ZY, YL, and YG wrote the manuscript. YG, JL, SS, and IS revised the manuscript. All authors contributed to the article and approved the submitted version.

## Funding

This work was supported by the Department of Science and Technology of Guizhou Province, China ([2020]1Y106, ZK[2021]YIBAN110, Qiankehezhicheng[2018]2344 and Qiankeheping tairencai[2020]6016), the National Natural Science Foundation of China (31660544, 32160648), the Major Science and Technology Program of China National Tobacco Corporation, China [11020200027(JY-10) and 110202101032(JY-09)], the Science and Technology Program of Guizhou Provincial Tobacco Company, China (201801, 2020XM02, 2020XM06, 2021XM05, 2021XM12, and 2022XM05), the Science and Technology Program of Zunyi Tobacco Company, China (2021XM05), the Sino-Pakistan Project NSFC (31961143008), the National Natural Science Foundation of China, International (Regional) Cooperation and Exchange Program, Research fund for International Young Scientists (31750110462) and Jiangsu Collaborative Innovation Center for Modern Crop Production (JCIC-MCP), China.

## Conflict of interest

The authors declare that the research was conducted in the absence of any commercial or financial relationships that could be construed as a potential conflict of interest.

## Publisher’s note

All claims expressed in this article are solely those of the authors and do not necessarily represent those of their affiliated organizations, or those of the publisher, the editors and the reviewers. Any product that may be evaluated in this article, or claim that may be made by its manufacturer, is not guaranteed or endorsed by the publisher.
